# Voice Interface Technology Adoption by Patients With Heart Failure: Pilot Comparison Study

**DOI:** 10.2196/24646

**Published:** 2021-04-01

**Authors:** Lida Anna Apergi, Margret V Bjarnadottir, John S Baras, Bruce L Golden, Kelley M Anderson, Jiling Chou, Nawar Shara

**Affiliations:** 1 Robert H. Smith School of Business University of Maryland College Park, MD United States; 2 Institute for Systems Research University of Maryland College Park, MD United States; 3 Georgetown University Washington, DC United States; 4 Medstar Health Research Institute Hyattsville, MD United States

**Keywords:** heart failure, telehealth, voice interface, conversational agent, artificial intelligence, wireless technology, social determinants of health, mobile phone

## Abstract

**Background:**

Heart failure (HF) is associated with high mortality rates and high costs, and self-care is crucial in the management of the condition. Telehealth can promote patients’ self-care while providing frequent feedback to their health care providers about the patient’s compliance and symptoms. A number of technologies have been considered in the literature to facilitate telehealth in patients with HF. An important factor in the adoption of these technologies is their ease of use. Conversational agent technologies using a voice interface can be a good option because they use speech recognition to communicate with patients.

**Objective:**

The aim of this paper is to study the engagement of patients with HF with voice interface technology. In particular, we investigate which patient characteristics are linked to increased technology use.

**Methods:**

We used data from two separate HF patient groups that used different telehealth technologies over a 90-day period. Each group used a different type of voice interface; however, the scripts followed by the two technologies were identical. One technology was based on Amazon’s Alexa (Alexa+), and in the other technology, patients used a tablet to interact with a visually animated and voice-enabled avatar (Avatar). Patient engagement was measured as the number of days on which the patients used the technology during the study period. We used multiple linear regression to model engagement with the technology based on patients’ demographic and clinical characteristics and past technology use.

**Results:**

In both populations, the patients were predominantly male and Black, had an average age of 55 years, and had HF for an average of 7 years. The only patient characteristic that was statistically different (*P*=.008) between the two populations was the number of medications they took to manage HF, with a mean of 8.7 (SD 4.0) for Alexa+ and 5.8 (SD 3.4) for Avatar patients. The regression model on the combined population shows that older patients used the technology more frequently (an additional 1.19 days of use for each additional year of age; *P*=.004). The number of medications to manage HF was negatively associated with use (−5.49; *P*=.005), and Black patients used the technology less frequently than other patients with similar characteristics (−15.96; *P*=.08).

**Conclusions:**

Older patients’ higher engagement with telehealth is consistent with findings from previous studies, confirming the acceptability of technology in this subset of patients with HF. However, we also found that a higher number of HF medications, which may be correlated with a higher disease burden, is negatively associated with telehealth use. Finally, the lower engagement of Black patients highlights the need for further study to identify the reasons behind this lower engagement, including the possible role of social determinants of health, and potentially create technologies that are better tailored for this population.

## Introduction

### Background

Heart failure (HF) is a condition in which a patient’s heart is unable to pump enough blood and oxygen to the organs. HF has high prevalence, affecting over 26 million people worldwide, and is associated with high mortality and health care utilization [1]. In the United States, there are currently 6.2 million adults with HF. Owing to the aging population, the number of individuals with HF is expected to exceed 8 million by 2030 (corresponding to approximately 2.97% of the US adult population) [2]. The total medical and indirect costs associated with HF are estimated to reach US $70 billion by 2030 [3]. Currently, in the United States, there are approximately 800,000 annual hospitalizations for the primary diagnosis of HF, and after each hospitalization, the 28-day and 1-year mortality rates are 10.4% and 29.5%, respectively [2]. Thus, it is critical to support patients with HF in managing their conditions once they are discharged from the hospital.

The long-term management of HF is closely associated with self-care. In addition to taking medications, patients are advised to reduce salt and fluid intake [4]; monitor their weight daily; stay active through appropriate physical activity; and evaluate potential signs and symptoms such as swollen ankles, weight increase, or shortness of breath [5].

Telehealth offers potential benefits for patients with HF because it allows their health care providers to collect daily patient feedback and, therefore, enables them to promptly intervene when necessary. Several telehealth approaches to HF have been examined. Structured telephone monitoring allows patients to answer a set of prerecorded questions regarding their symptoms through their telephone keypad [6]. Increased internet access has further enabled the development of numerous technologies [7-11], with some examples presented later. Patients can log on to a designated website to enter information about their daily symptoms, which allows nurses to monitor any changes [11]. Through an Xbox gaming platform, patients with HF can navigate through screens, answer multiple-choice questions regarding their symptoms, read further instructions about their self-care, and learn more about their condition [8]. Tablets connected to a weight scale and a blood pressure wrist monitor allow patients to send daily readings to their health care provider [10]. Similarly, a number of recent studies have investigated smartphone apps that can be used by patients with HF to submit daily symptoms, transmit vital readings, and receive feedback about their health [7,12-16].

A recent review of studies on telehealth adoption by patients with HF can be found in the study by Gorst et al [17]. Across the studies discussed, the main factors identified as negative influences on telehealth adoption include difficulties with using the required technology, not remembering to use the technology every day, and considering the telehealth procedure to be redundant or boring. Therefore, it can be concluded that a critical component of any telehealth application is its ease of use and engagement. Conversational agent technology using a voice interface is a potential solution because it can ask patients questions through speech and understand their answers through speech recognition.

Conversational agents have been used in numerous health care settings, with the literature extensively focusing on mental health applications [18,19]. However, conversational agent technology using a voice interface has also been used to help support behavior change and promote a healthy lifestyle [20]. Furthermore, in a few studies, the technology has been recommended for patients with HF to collect information about symptoms and management of their conditions. In particular, the proposed designs for voice interface technology for patients with HF can be found in the studies by Ferguson et al [21] and Zhang et al [22]. However, these studies did not provide results for evaluating the implementation of the proposed technology. One study on a small cohort of patients with HF, investigating the satisfaction and engagement with conversational agent technology, found high user satisfaction and an average engagement of about 60% [23]. However, the technology in this study was a chatbot that did not have a voice interface. Furthermore, this study had a small number of participants (5 patients) and did not examine how the characteristics of the patients impacted their level of engagement.

### Objectives

In this paper, we investigate which patient characteristics are associated with patient engagement with the voice interface technologies. To the best of our knowledge, this is the first study to examine the factors that influence HF patients’ adoption of voice interface technology.

## Methods

### Voice Interface Technologies

We studied a voice technology using two different user interfaces, Alexa+ and a visual Avatar (both introduced in detail later). Data for the two technologies were collected from two different studies. The study of the Alexa+ technology was funded by the National Institutes of Health (trial registration number: NCT03707275), and the study for the Avatar was investigator initiated and industry funded. The two studies followed the exact design and protocol, which enabled us to compare user engagement with the 2 different voice interfaces and, more generally, to compare the drivers of their use.

Alexa is a virtual assistant artificial intelligence technology developed by Amazon. Alexa is voice activated and has a number of functions, including sending messages, playing music, and providing traffic updates. For this study, the Alexa Skills Kit was used to expand the original capabilities of Alexa technology. A voice-activated survey was developed that enabled patients to answer a series of questions regarding their conditions and receive feedback. The resulting technology (Alexa+) was implemented using Echo Dot devices, which are smart speakers that can be used to access Alexa. The retail price for an Echo Dot is US $50 per unit; thus, this technology is relatively affordable.

**Figure 1:**
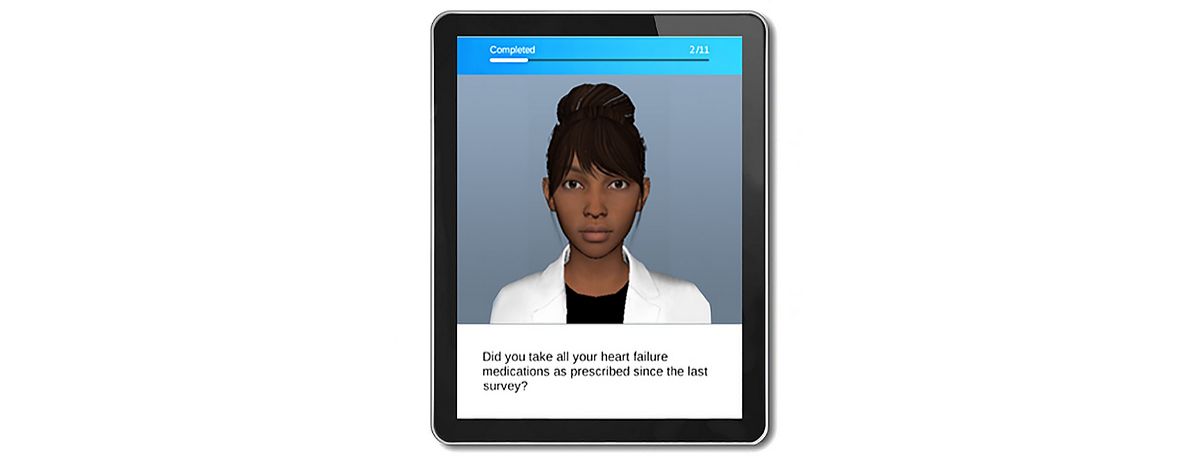
Avatar on a tablet.

The Avatar interface was developed by Oben [24]. The appearance of the Avatar was designed to be both reflective of the care team from the patient’s hospital and have characteristics of the patient population, where a majority of our patients were African Americans. The Avatar was programmed to ask the patients the same series of questions related to their HF treatment and symptoms and provide feedback. The Avatar app was saved on tablets that had no other apps. The avatar used in this study is shown in Figure 1.

Note that in addition to the way that information is presented, one difference between the two technologies is that the Alexa+ technology allows patients to set up daily reminders. Thus, patients participating in the Alexa+ study had the option to receive reminders at a particular time of day, which prompted them to answer the questionnaire. However, the tablets used in the Avatar study did not have this option.

Both Alexa+ and Avatar were populated with the same script. The detailed script, including the questions asked, their order, and the comments that the voice interfaces make in each case, can be found in Multimedia Appendix 1. In the first stage of the script, the voice interface asked the patients with HF 11 questions, which can potentially be expanded to 13 questions, depending on the answers given by the patients. Patients answered each question separately, with yes or no. The answer that the patient provides to each question affects the type of comment or/and interjection that the voice interface makes right after the answer, as well as the next question. The 11-item questionnaire was divided into three components: compliance (questions 1-3), mild HF symptoms (questions 4-6), and moderate or severe HF symptoms (questions 7-11). The answers were captured, color coded, and displayed using a Tableau dashboard developed specifically for this study. The colors reflect the risk level of patients associated with each of the 3 components. The compliance questions evaluated whether a patient weighed themselves, took their HF medication, and avoided eating high-salt food. A negative answer to any of these questions raises a blue flag for that question. The mild HF symptom questions were designed to determine if the patient experienced shortness of breath with regular activity, had a cough, or had swollen ankles. If the patient gives a positive answer to any of the mild HF symptom questions, it raises an orange flag for the corresponding question. The moderate or severe HF symptom questions have to do with weight increase and shortness of breath at rest or while sleeping. If the patient answered yes to any of the moderate or severe HF symptom questions, a red flag was raised. The raised flags can result in alerts, and any red flag affects the tailored response that the voice interface gives the patient once the questionnaire is completed. This is done in the second stage of the script, where the voice interface summarizes the answers provided by the patient and then provides the response.

The HF patient survey contained within the script was created based on the existing literature, including the literature on HF action plans, which were used for self-care management and symptom recognition [25-27], and the literature on previously developed telehealth systems that can help with the management of HF [28,29]. Color-coded alerts based on participants’ answers were previously used in a telemanagement pilot study [28], where the system provided feedback based on a three-zone action plan. The three zones consisted of green (*stay in this zone*), yellow (*warning*), and red (*seek immediate help*). Similar color-coded zones were used in the studies by Vincent and Mutsch [30,31], where the proposed action plan consisted of green, yellow, and red zones. The green zone indicated that the patient was doing well, and patients were instructed to continue current medications, diet, and activities. The yellow zone indicated caution, and patients were advised to follow a low-sodium diet, take prescribed medications, and take an extra diuretic dose if necessary. The red zone indicated a medical emergency, and the patient was instructed to immediately obtain help.

### Patient Enrollment and Training

A total of 30 patients were enrolled in each of the two studies using the same eligibility criteria. All participants had at some point in the past either been admitted to the MedStar Washington Hospital Center for HF or had been seen in a MedStar Heart Failure Clinic for HF. Furthermore, the patients were required to be aged 18 years or older and live in a house with Wi-Fi access. Finally, the patients could not participate in the studies if they had had a heart transplant or if they had a ventricular assist device. Participation was voluntary; patients who declined the offer to participate provided different reasons, including not wanting to take the daily surveys, not wanting another device in their lives, or lack of interest.

Participants did not receive any monetary incentives for participation; however, they were allowed to keep the Alexa and tablet devices after their participation in the study was completed. Participants were identified via electronic health records (EHRs) and by reviewing the schedules of providers at the aforementioned cardiology clinics. When potentially eligible participants were identified, the study coordinator introduced the study to them, discussed the details and logistics of the study and the risks and benefits, and allowed the participants to take time to make an informed decision about whether to participate. If they decided to take part, they signed the informed consent document, medical history information was collected, and the coordinator then proceeded with assigning them the corresponding technology.

Participants in the Alexa+ study were recruited from December 2018 to March 2019 and were provided a study-specific Amazon account, equipped with an Echo Dot configured to access the Alexa+ app. Participants were provided training on the Alexa Echo Dot, including Alexa voice training. The voice training consisted of a session of 25 phrase repetitions that allowed Alexa to improve its voice recognition capabilities for the target user. The patients participating in the Avatar study were recruited from February to December 2019 and were provided tablets with the Avatar app and were shown how to use the technology. In addition, during their training, patients participating in both studies completed their first questionnaire to ensure that the device was working properly and to answer any questions that the patients may have had.

### Demographic and Technology Survey

Before patients started using the technology that was assigned to them, they completed a demographic and technology survey. This allowed us to identify patient characteristics associated with low- or high-voice interface technology adoption in subsequent analyses. In particular, the demographic section of the survey included questions about age, gender, marital status, race, Hispanic heritage, annual household income, education, insurance coverage, number of years with HF, number of medications to manage HF, and visual impairment. The technology section of the survey included questions about the type of mobile phone, whether they used their phone to send text messages, whether they accessed social media and browsed the internet on their phones, and how confident they felt using computers or other electronic devices.

### Monitoring

**Figure 2:**
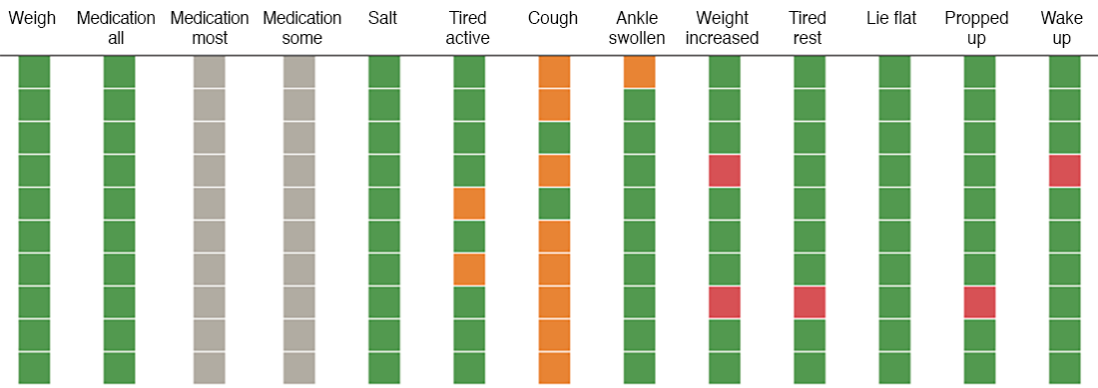
Example of color display used in the monitoring of patients, as it appears in the email sent to the study nurse. Green indicates that the patient either complied with the corresponding instruction (columns 1-5) or did not have the corresponding symptom (columns 6-13), orange indicates that the patient gave a positive answer to the corresponding mild heart failure (HF) symptom question, and red indicates that the patient gave a positive answer to the corresponding moderate or severe HF symptom question. Gray indicates skip logic, meaning these questions were not required based on the previous answers.

Once enrolled, patients were instructed to complete the questionnaire daily for 90 days. For participants who answered in a manner that indicated HF stability, the response was coded green and no alerts were generated. As discussed earlier, patients’ responses could be deemed clinically undesirable if they raised any blue (compliance questions), orange (mild HF symptom questions), or red (moderate or severe HF symptom questions) flags. Red-flagged questions generated immediate text and email alerts to the study nurse, who monitored the alerts daily, including weekends and holidays. The texts contained the participant identification number and the following alert: “We have received a concerning daily response from patient [PatientID] that warrants your attention: [QuestionID] yes.” In total, 281 alerts were generated for the Alexa+ group and 404 for the Avatar group. In each email alert, the answers were also summarized as a color display on the dashboard (Figure 2). Note that the gray color in the figure indicates skip logic, meaning these questions were not required based on the previous answers. Both questions have to do with medication compliance (Multimedia Appendix 1).

For each patient, a baseline was established based on their initial responses to the questionnaire. The study nurse reviewed alerts daily and evaluated each participant’s stability compared with their baseline; any changes were evaluated based on each patient’s initial answers.

Certain information gathered through our study was shared with the staff of the study institution. As part of the institution’s general care practices, each patient with HF is assigned a nurse navigator, who is responsible for coordinating the clinical care of the participant. The nurse navigator at the study institution, the principal investigator, and the study physician were all informed of any change in the status of the patient. A change in status was defined as either a change from baseline in moderate or severe HF symptoms, multiple red flag responses, or persistent red flag responses.

Every 3 weeks, the study coordinator would contact participants who did not complete the questionnaire to check on the participant’s status, reemphasize the importance of using the technology, and encourage completion. If needed, the study coordinator would provide additional training to the participants on how to initiate and communicate with the device. In some cases, participants would call and inform the study coordinator that they were traveling out of the state or country or on vacation and were unable to complete the questionnaire. 

### Analysis

We used descriptive statistics to characterize the demographic and clinical characteristics of the patients and their experience and confidence in using technology. In particular, we estimated the means and SD for the continuous variables and counts and corresponding percentages for the categorical variables. Furthermore, we report the number of missing values for each variable. We compared the patient populations participating in the Alexa+ study with that of the Avatar study based on the aforementioned characteristics. We used chi-square tests to compare the counts in the different categories between the two populations for each of the 12 categorical variables, and we used 2-sample *t* tests to test the differences between the two population means for the 3 continuous variables. We report the corresponding *P* values for each variable.

This analysis is based on data from demographic and technology surveys. However, in some cases, data regarding demographic and clinical characteristics were also retrieved from patients’ EHRs to fill in missing values.

We defined the variable of interest in our study, patient engagement, as the number of days during the study period in which the patient used the corresponding voice interface to answer the questionnaire. We chose this variable because daily engagement with the technology is important for patients with HF, as their condition can deteriorate quickly. For example, weight gain overnight can be a sign of fluid retention. To capture such rapidly changing clinical signs and to promote successful self-care and health monitoring, patients were asked to use the technology daily. Our use of the number of days of compliance is similar to that in other studies in the literature [15,32]. We note that using the percentage or portion of days as the variable of interest would result in identical results and insights, as the study period is fixed. In addition, using the number of days facilitates easy interpretation of the regression model. We used visualizations to demonstrate how patient engagement changed over time in each study and to obtain an initial estimate of engagement levels.

We modeled the relationship between the engagement level and the patients’ characteristics using multiple linear regression on the entire population (combining both studies). The dependent variable is the number of days that the patient used the voice interface technology during their own specific 90-day period. The independent variables include the demographic, clinical, and technology-related characteristics of the patient. In particular, we generated a binary variable for patients who had a smartphone, for patients who were very confident in using technology, for patients who had only a little or no confidence in using technology, for patients who had a college education or higher, for patients who had a high school education or less, for patients with an annual household income higher than US $100,000, for patients with an annual household income less than US $25,000, for patients who were married, for patients who were identified as Black, and for patients who were identified as White. Finally, to differentiate between the two studies, we generated a binary variable, taking the value of 1 for patients who were enrolled in the Avatar study and the value of 0 for patients enrolled in the Alexa+ study.

Given the small sample size, the variables were carefully selected. We chose to use the best subset approach to generate a model that explains the variation in the patient’s engagement as well as possible while using as few variables as possible. In all, 15 control variables were measured at the time of enrollment. We noted that although the data were not normalized, all of the control variables were binary, with the exception of the number of medications used, years with HF, and age. We used the adjusted *R*^2^ value for model selection. Like any reduced model, the interpretation of the coefficients may be biased if important variables are excluded from the model. A follow-up sensitivity analysis was conducted to help us understand the interaction between the type of technology and income level.

## Results

### Population Statistics

Of the 30 patients, 3 initially enrolled in the Avatar study were subsequently withdrawn because they could not be reached after they provided their initial participation consent. Table 1 shows the demographic, clinical, and technology-related characteristics of the patients participating in each study. As shown in the table, patients participating in the Alexa+ study were taking significantly more medications to manage their HF, with a mean of 8.7 (SD 4.0), as compared with patients in the Avatar study, with a mean of 5.8 (SD 3.4; *P*=.008). For the remaining variables, there were no statistically significant differences between the two patient populations.

Overall, both populations were predominantly male (60% with Alexa+ and 63% with Avatar), Black (60% with Alexa+ and 63% with Avatar), had an average age of approximately 55 years (mean age 54 years, SD 11.7 for Alexa+ and 56.5 years, SD 12.1 for Avatar), and had had HF for an average of about 7 years (mean of 7.5, SD 8.1 for Alexa+ and 7.3, SD 6.4 for Avatar). Furthermore, the majority of patients in both studies had experience using smartphones and had confidence in using similar technology.

**Table 1:** Characteristics of patients participating in the two studies.

Characteristic		Alexa+ (n=30)	Avatar (n=27)	*P* value
**Age (years), mean (SD)**		54.0 (11.7)	56.5 (12.1)	.45
	Missing, n (%)	2 (7)	1 (4)	
**Gender, n (%)**				>.99
	Male	18 (60)	17 (63)	
	Female	10 (33)	10 (37)	
	Missing	2 (7)	0 (0)	
**Marital status, n (%)**				.80
	Single, never married	7 (23)	6 (22)	
	Married	11 (37)	15 (56)	
	Living together, not married	3 (10)	1 (4)	
	Separated or divorced or widowed	6 (20)	5 (19)	
	Missing	3 (10)	0 (0)	
**Race, n (%)**				.54
	Black	18 (60)	17 (63)	
	Asian	0 (0)	1 (4)	
	White	7 (23)	8 (30)	
	Other	4 (13)	1 (4)	
	Missing	1 (3)	0 (0)	
**Hispanic heritage, n (%)**				>.99
	Yes	1 (3)	0 (0)	
	No	27 (90)	27 (100)	
	Missing	2 (7)	0 (0)	
**Annual household income (US $), n (%)**				.17
	0-25,000	10 (33)	5 (19)	
	25,001-50,000	9 (30)	4 (15)	
	50,001-100,000	2 (7)	5 (19)	
	More than 100,000	5 (17)	8 (30)	
	Missing	4 (13)	5 (19)	
**Education level, n (%)**				.49
	Some high school or high school graduate	11 (37)	9 (33)	
	Some college	10 (33)	7 (26)	
	College graduate	3 (10)	2 (7)	
	Postgraduate degree	2 (7)	6 (22)	
	Missing	4 (13)	3 (11)	
**Years with HF^a^, mean (SD)**		7.5 (8.1)	7.3 (6.4)	.95
	Missing, n (%)	5 (17)	8 (30)	
**Number of medications to manage HF, mean (SD)**		8.7 (4.0)	5.8 (3.4)	.008
	Missing, n (%)	2 (7)	1 (4)	
**Visually impaired or blind, n (%)**				.11
	Yes	4 (13)	0 (0)	
	No	22 (73)	25 (93)	
	Missing	4 (13)	2 (7)	
**Type of mobile phone, n (%)**				.41
	Basic	1 (3)	2 (7)	
	Smart	25 (83)	22 (82)	
	None	0 (0)	1 (4)	
	Missing	4 (13)	2 (7)	
**Uses phone to text, n (%)**				>.99
	Yes	25 (83)	24 (89)	
	No	1 (3)	1 (4)	
	Missing	4 (13)	2 (7)	
**Uses phone for social media, n (%)**				.58
	Yes	15 (50)	17 (63)	
	No	11 (37)	8 (30)	
	Missing	4 (13)	2 (7)	
**Uses phone to browse the internet, n (%)**				.23
	Yes	24 (80)	20 (74)	
	No	2 (7)	5 (19)	
	Missing	4 (13)	2 (7)	
**Confidence in using technology, n (%)**				.87
	Very	11 (37)	12 (44)	
	Somewhat	13 (43)	10 (37)	
	Only a little	2 (7)	2 (7)	
	Not at all	0 (0)	1 (4)	
	Missing	4 (13)	2 (7)	

^a^HF: heart failure.

### Technology Engagement

A few patients did not complete the study. In the Alexa+ study, 5 patients withdrew from the study, 2 patients had issues with their Wi-Fi connection, and 2 patients did not respond to contact attempts. Furthermore, 1 participant in the Avatar study did not respond to any contact attempts. Thus, from the Alexa+ study and the Avatar study, we obtained technology use information for 21 out of the 30 patients and 26 out of the 27 patients, respectively. The resulting study cohort is, therefore, 47 patients.

**Figure 3:**
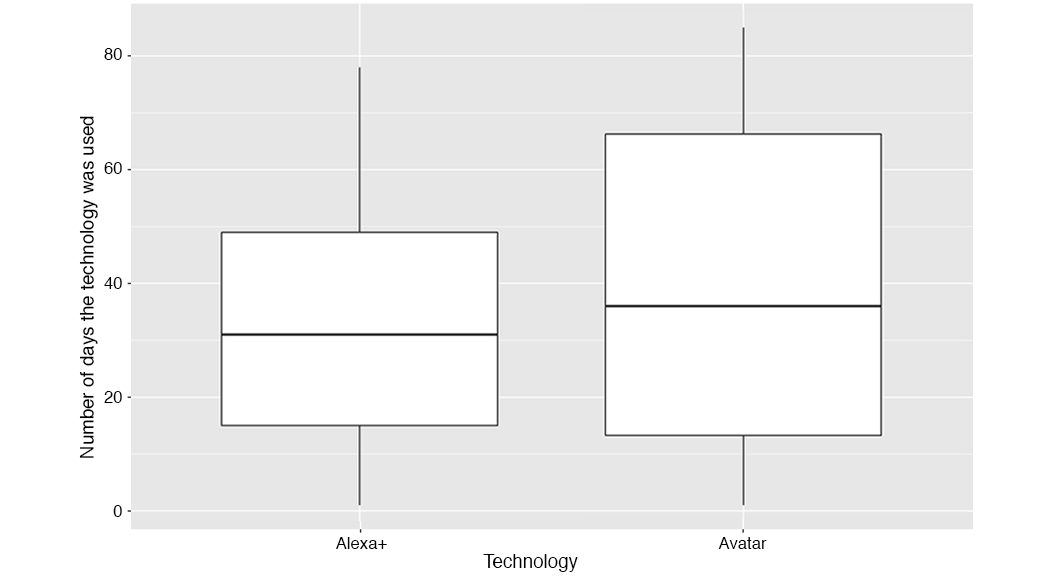
Box plots showing the number of days that the patients interacted with the voice interface in each study.

Alexa+ patients used the technology a mean of 35.3 times (SD 26.0), whereas the Avatar patients used it a mean of 37.8 times (SD 28.9). The *t* test of the difference between the two population means had a *P* value of .76, indicating that the difference in the engagement levels between the two groups was not statistically significant. Figure 3 highlights the variations in the level of engagement of patients participating in each study. For both technologies, we observed a large range of values in the number of times that patients interacted with the voice interface during the 90-day period. Although there were some patients in both studies who engaged with the technology almost daily, significantly less use of the technology was seen among the majority of participants.

Regarding the engagement over time for the two technologies, we observe a decrease in use over time. Multimedia Appendices 2 and 3 show the use over time for the two technologies. The decrease in use was sharper for Alexa+ participants. On the other hand, participants in the Avatar study demonstrated more stable technology use in the second half of the study period.

### Regression Results

The regression model (Table 2) had an adjusted *R*^2^ of 28.13%. The resulting coefficients indicate that higher patient age is linked to higher use of the technology (1.19; *P*=.004), whereas Black patients used the technology fewer times than non-Black patients with otherwise similar characteristics (−15.96; *P=*.08). Excluding other race indicator variables, the variable for the Black race had a relatively strong correlation with the variable showing that the patient was married (−0.52) and moderate correlation with an annual household income less than US $50,000 (0.24) and years with HF (−0.22). With the remaining variables, there were weaker correlations. Thus, this large difference in engagement of Black patients may be an indication of other important socioeconomic or medical factors or other confounding factors that are beyond the scope of our data.

**Table 2:** Linear regression model for predicting the number of times that the patient used the voice interface technology.

Variable	Coefficient	95% CI	*P* value
Intercept	17.93	−27.71 to 63.58	.43
Age	1.19	0.42 to 1.96	.004
Black	−15.96	−33.84 to 1.92	.08
Household income higher than US $100,000	12.28	−5.49 to 30.05	.17
Confidence in using technology	12.88	−3.66 to 29.42	.12
Number of medications to manage HF^a^	−5.49	−9.22 to −1.72	.005
Avatar study participant	−24.14	−44.29 to −3.98	.02

^a^HF: heart failure.

The model also shows that patients who take a higher number of HF medications tended to demonstrate lower use of the technology (−5.49; *P*=.005). Finally, patients who participated in the Avatar study interacted less with the technology (−24.14; *P*=.02) compared with patients with similar characteristics who participated in the Alexa+ study. The direction of the Avatar regression coefficient is surprising when compared with Figure 3, which shows that the use of Avatar technology is higher on average. Therefore, we conducted a sensitivity analysis to understand what drives the coefficient value.

### Sensitivity Analysis

**Figure 4:**
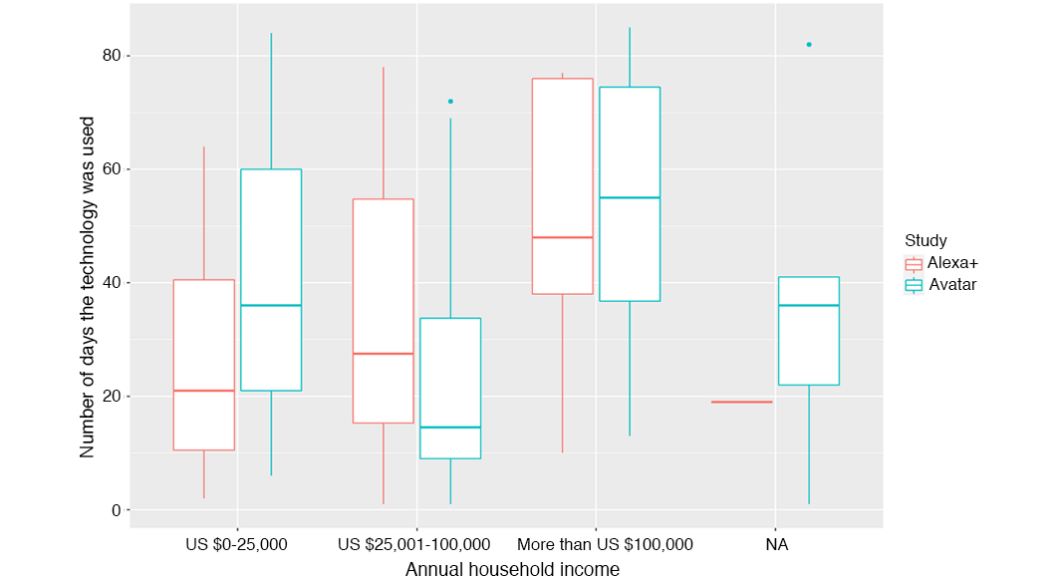
Box plots showing the number of interactions in each study based on household income. NA: income information not available.

To further compare the participation levels in the Avatar and Alexa+ studies, we stratified participation by income levels. Figure 4 shows the engagement levels for each technology as a function of the income level. Note that the last group of boxplots corresponds to observations that have a missing value for household income. We see that although engagement with the technology increases as the household income increases in the case of the Alexa+ participants, the participation levels appear different for the Avatar participants. In particular, participants with a household income between US $25,000 and US $100,000 showed lower engagement than comparable patients in the Alexa+ study. As income appears to have a different influence on engagement with the two technologies, we added interaction terms between Avatar and the different income levels and then reran the best subset regression.

The resulting regression model is shown in Table 3. The same overall patterns hold. Higher patient age was linked to higher use of the technology (1.07; *P*=.004), whereas Black patients used the technology less frequently (−21.35; *P*=.02). Again, patients who take a higher number of medications to manage HF use the technology less on average (−6.52; *P*=.002).

From the model, we see that, overall, Avatar patients with middle- and high-income levels are expected to interact less with the technology compared with otherwise similar Alexa+ patients (−32.38; *P*=.006). However, the best subset regression retained the interaction term between low income and Avatar, which, together with the Avatar coefficient, indicates that, on average, those from lower income households use Avatar technology more than otherwise similar Alexa+ participants. The interaction coefficient equals 44.81 (*P*=.04), which, combined with the Avatar coefficient, estimates that Avatar participants from low-income households engage over 12 times more with the technology than similar Alexa+ participants. In other words, with everything else held constant, the model estimates that low-income patients use Avatar more often than Alexa+ technology, whereas the impact is reversed for middle-income patients and higher income patients. The overall impact of high and low household income was not statistically significant.

**Table 3:** Linear regression model for predicting the number of times that the patient used the voice interface technology, with an added interaction term.

Variable	Coefficient	95% CI	*P* value
Intercept	43.45	2.54 to 84.36	.04
Age	1.07	0.37 to 1.78	.004
Black	−21.35	−39.19 to −3.50	.02
Household income higher than US $100,000	15.19	−2.87 to 33.24	.10
Household income lower than US $25,000	−3.89	−26.61 to 18.83	.73
Number of medications to manage HF^a^	−6.52	−10.33 to −2.70	.002
Avatar study participant	−32.38	−54.70 to −10.06	.006
Avatar×income lower than US $25,000	44.81	2.44 to 87.18	.04

^a^HF: heart failure.

## Discussion

### Principal Findings

In this study, we compared the engagement levels of patients with HF with two different voice technology interfaces. To enable such a comparison between the two groups, the design and patient enrollment of both studies and the setup of both technologies were identical. Although the study was based on a small number of patients, the regression analysis helped identify some key characteristics that are linked to different engagement levels, contributing to the growing literature on technology use for chronic disease management [33].

On the basis of our analysis, Black patients used the technology 21 fewer times on average during the study period compared with non-Black patients with otherwise similar characteristics. As in our data set, Black race is not highly correlated with other features, this study adds to the evidence that technology design may need to be better tailored for this population [14]. Although our findings contribute to the emergent and growing literature on racial disparities in technology use, this area is still understudied. For instance, a recent survey on self-management among patients with HF points to only 4 studies that evaluate the use of technology for health management among Black patients with HF [34], the population that is arguably at the highest risk. Further examination to shed light on why Black patients engage less with health management technology is much needed, including the potential role of social determinants of health.

In addition, we see that patients taking a higher number of medications to manage HF interact less on average with the technology. The number of medications can be interpreted as a proxy for the severity of the HF condition or an indication of additional comorbid conditions. However, we note that the literature on the impact of disease burden on patient technology and self-care engagement is mixed; some studies have previously found that patients’ willingness to self-monitor is not directly related to their health problems [35], whereas other studies indicate that sicker patients are more actively engaged [36] or less actively engaged [16,37]. The use of the number of medications for HF as a proxy for the severity of the condition has obvious limitations. A more direct measure of the severity of HF (eg, the New York Heart Association functional class [38] or the American College of Cardiology and American Heart Association stages of HF [39]) would be needed to more directly study the connection between patient engagement and disease severity in patients with HF.

Finally, our study finds that older patients tend to exhibit higher levels of engagement with voice interface technologies. This agrees with findings from previous studies that patient age affects the level of adherence, with older patients using telemonitoring more regularly [15,40-42].

### Future Research

The results presented here encourage future study of multiple aspects of voice interface technology, including its varying impact on and its potential to empower patients who are affected by different diseases. Specifically, important work can be done by asking how best to operationalize these technologies and adapt them to different populations to support engagement. For example, one avenue for future work lies in advancing the technology’s feedback: useful and personalized feedback is known to increase the level of engagement, and as a recent study points out, “The key to successful technology-based treatment is ease to use in a personalized manner such that ongoing feedback can be incorporated into these technology-based tools that keeps patients engaged” [43]. Although the technology used in this study did adjust its feedback in response to patient answers, more can be done to personalize such feedback. Future advances may draw on natural language technology, including voice interface technology, which is evolving quickly. Although this study used preset questions and feedback based on each patient’s answers, the future of voice-assisted health monitoring may lie not in simple rule-based questions and answers but in open-ended questions to which a conversational agent can respond. In addition, it may be possible to better personalize voice interface technologies; for instance, drawing on the rich medical history data in EHR would allow us to create personalized voice interface technologies tailored to the specific patient’s disease and preferences. Other potential avenues for increasing engagement include explaining the benefits of the technology and the details of its use. The engagement levels observed in this study highlight the importance of encouraging patients to use the technology by increasing awareness of its role in helping them manage their condition.

Our results further show the importance of future research into patient health factors associated with low levels of utilization and those that drive patient engagement. Further studies on larger sets of patients would allow for a more in-depth study of such patient engagement drivers, including the interaction between socioeconomic status and engagement levels. Such work could inform the development of voice interface systems, as this process should take patients’ preferences, socioeconomic factors, and other population-level considerations into account. As a result, the potential benefits of voice interface apps can be more effectively leveraged for individuals and groups who are often underrepresented. In addition, larger studies of engagement drivers could allow for a better comparison between the drivers of engagement with voice interfaces and the drivers of engagement with other modes of self-monitoring. In general, a broader and deeper understanding of physiological and psychological factors that may prevent a patient from using a specific kind of technology would further the potential of that technology for patient self-management and early alerts of worsening condition.

This pilot study highlights the potential of patient engagement with a voice interface to help patients better manage their health; in addition to the larger issues of patient engagement discussed earlier, it opens up other future research possibilities. First, as this study compares two modes of voice technology, it does not shed light on the benefit of a voice interface over other self-monitoring technologies. In addition, this study was limited to patients with HF, whose daily self-monitoring is important (as, eg, change in weight overnight can be a sign of serious complication). Daily self-monitoring is also crucial for the management of many other chronic diseases, and the extent to which our results transfer to other such chronic disease contexts is important and worthy of future study.

### Conclusions

HF is a chronic condition that requires sustainable management. Voice interface technologies present an opportunity to empower patients to better manage their health by setting reminders and following condition-specific instructions to potentially prevent hospitalizations and emergency department visits. Our results show that an easy-to-use voice interface may play a significant role in helping older patients manage their chronic conditions and enhance remote engagement between patients and their providers to prevent worsening of the patient’s condition. These innovative solutions have the potential to be scaled to address other chronic and more acute conditions and identify which subgroups of patients may best benefit from these technologies.

## Appendix

Multimedia Appendix 1 Voice interface scripts.

Multimedia Appendix 2 Percentage of Alexa+ participants (n=21) who logged in during each day in the study.

Multimedia Appendix 3 Percentage of Avatar participants (n=26) who logged in during each day in the study.

## Abbreviations

EHR: electronic health record
HF: heart failure
