# Supplementary material for: Voice Interface Technology Adoption by Patients With Heart Failure: Pilot Comparison Study
Source: JMIR Mhealth Uhealth. 2021 Apr 1;9(4):e24646. doi: 10.2196/24646 (PMC8050751; doi:10.2196/24646)
Supplement: Multimedia Appendix 1 [file mhealth_v9i4e24646_app1.docx]

## Multimedia Appendix 1: Voice interface scripts

Tables 1 and 2 include the script followed by the voice interfaces. In particular, Table 3 includes the questions asked of the patient, and the corresponding action of the voice interface based on the patient’s answer (i.e., what to comment, and which question to ask next). Table 4 includes the comments that the voice interface makes to the patient after the questionnaire is completed.

Table 1: Script followed by both technologies. Part 1: Questions asked.

| QID | Question | Patient’s  Answer | Comment [& Action] |
| --- | --- | --- | --- |
| 1 | Did you weigh yourself today? | Yes | * Great, then. [Ask Q 2a]  * Excellent. And [Ask Q 2a]  * Okay! [Ask Q 2a]  * Good. And [Ask Q 2a]  * Wonderful. Now, [Ask Q 2a] |
|  |  | No | * Ok - Please weigh yourself after you complete this survey [Ask Q 2a]  * Got it. Please weigh yourself every after you finish this survey. [Ask Q 2a]  * Ok, remember to weigh yourself after you finish this survey. And [Ask Q 2a]  * It's important to weigh yourself every day. Please weigh yourself after you complete this survey. Now, [Ask Q 2a] |
| 2a | Did you take all your heart failure medications as prescribed since the last survey? | Yes | * Wonderful, [Ask Q 3]  * Good to hear. [Ask Q 3]  * Great. [Ask Q 3]  * Good! [Ask Q 3]  * Okay, [Ask Q 3] |
|  |  | No | [Ask Q 2b] |
| 2b | Did you take most of your heart failure medications as prescribed since the last survey? | Yes | * Okay. Please be sure to always take all your medications as prescribed. [Ask Q 3]  * Okay, but it’s important to take all your medications as prescribed every day. [Ask Q 3]  * Good to hear but please be sure to take all your medications exactly as prescribed every day. [Ask Q 3] |
|  |  | No | [Ask Q 2c] |
| 2c | Did you take some of your heart failure medications as prescribed since the last survey? | Yes | * Okay. Please be sure to always take all your medications as prescribed. [Ask Q 3]  * Okay, but it’s important to take all your medications as prescribed every day. [Ask Q 3]  * Fine but please be sure to take all your medications exactly as prescribed every day. [Ask Q 3] |
|  |  | No | * Please be sure to take your medications right after we finish this survey. [Ask Q 3]  * Please be sure to take your medications after you complete the rest of the survey. It's important to take your medications every day. [Ask Q 3]  * It is very important you take your medications exactly as prescribed.  Please do it after we are done with your heart health survey [Ask Q 3]  * Following your medication prescription is very important - please take your medications after you finish your heart health survey. [Ask Q 3] |
| 3 | Did you eat any high salt foods since the last survey? | No | * Keep it up, now [Ask Q 4]  * Good. And [Ask Q 4]  * Okay, [Ask Q 4]  * Great to hear! [Ask Q 4]  * Perfect. [Ask Q 4] |
|  |  | Yes | * Remember to keep your salt under 3 grams a day, and don't add additional salt to your food. [Ask Q 4]  * Please keep your salt intake under 3 grams a day, it’s important for your health. [Ask Q 4]  * Remember, no more than 3 grams of salt a day, and don't add extra to your food. [Ask Q 4]  * Your doctor recommends that you keep your salt under 3 grams a day, and refrain from adding extra salt at the table. [Ask Q 4] |
| 4 | Do you feel tired or short of breath with regular daily activities such as eating or taking a shower? | No | * Positive Interjection ^a^, [Ask Q 5] |
|  |  | Yes | * Negative Interjection ^b^, [Ask Q 5] |
| 5 | Do you have cough or wheezing? | No | * Positive Interjection ^a^, [Ask Q 6] |
|  |  | Yes | * Negative Interjection ^b^, [Ask Q 6] |
| 6 | Are your ankles swollen? | No | * Positive Interjection ^a^, [Ask Q 7] |
|  |  | Yes | * Negative Interjection ^b^, [Ask Q 7] |
| 7 | Did your weight increase by 3 pounds or more in 1 day? | No | * Positive Interjection ^a^, [Ask Q 8] |
|  |  | Yes | * Negative Interjection ^b^, [Ask Q 8] |
| 8 | Do you feel tired or short of breath at rest (for example when sitting | No | * Positive Interjection ^a^, [Ask Q 9] |
|  |  | Yes | * Negative Interjection ^b^, [Ask Q 9] |
| 9 | Do you feel short of breath when you lie flat in bed? | No | * Positive Interjection ^a^, [Ask Q 10] |
|  |  | Yes | * Negative Interjection ^b^, [Ask Q 10] |
| 10 | Do you need to be propped up with pillows in order to be able to sleep without shortness of breath? | Yes / No | * Okay, we’re almost done. [Ask Q 11]  * Almost there. [Ask Q 11]  * Final question. [Ask Q 11]  * Right. Last question, [Ask Q 11]  * And lastly, [Ask Q 11] |
| 11 | Do you wake up from sleep in the middle of the night with shortness of breath and need to sit up to feel less short of breath? | Yes / No | * Thank you for completing your heart health survey.  * You are all done with your heart health survey.  * You have completed your heart health survey for today. |

^a^ **Positive Interjections (Randomly chosen):** * Got it. * Okay. * All right! * Excellent. * That's good, * Fine. * Right. * Nice. * Very Good. * Oh good. * Great. * Good * That’s great * Wonderful

^b^ **Negative Interjections (Randomly chosen):** * Okay, * Right, * Okay, I see. * Oh, I see. * Got it. * Alright * I see. * Sorry to hear that * All right!

Table 2: Script followed by both technologies. Part 2: Advice given.

| Any Red Flags? | Conditions | Comment |
| --- | --- | --- |
| No | If “WEIGHED SELF” & “TOOK MEDS” & “NOT MUCH SALT” | * You did well, today, weighing yourself, taking your medications, and complying with heart failure diet  *Well done, today, you weighed yourself, took your medications, and complied with the heart failure diet  * Way to go, weighing yourself, taking your medications, and keeping your salt intake under 3gms, today. |
|  | “WEIGHED SELF” & “TOOK MEDS” | * Good job, today. You just need to keep your salt intake under 3gms  * You did well today. You just need to comply with the heart failure diet. |
|  | Else If “WEIGHED SELF” & “NOT MUCH SALT” | * Good job, today. You just need to take your medications as prescribed  * You did well, today. You just need to take your medications as prescribed to make it perfect |
|  | Else If “TOOK MEDS” & “NOT MUCH SALT” | * Good job, today. You just need to weigh yourself after we are done.  * You did well, today. You just need to weigh yourself after we are done to make it perfect |
|  | Else If “WEIGHED SELF” | * Please remember to take your medications when we are done, as prescribed, and to keep your salt intake under 3gms. |
|  | Else If “TOOK MEDS” | * Please remember to weigh yourself and record your weight when we are done, and to comply with the heart failure diet. |
|  | Else | * Please remember to weigh yourself and record your weight when we are done, and to take your medications as prescribed. |
| Yes | If “WEIGHED SELF” & “TOOK MEDS” & “NOT MUCH SALT” | // Do nothing |
|  | Else If “WEIGHED SELF” & “TOOK MEDS” | * Please make sure to keep your salt intake under 3gms  * Remember it is very important to comply with the heart failure diet. |
|  | Else If “WEIGHED SELF” & “NOT MUCH SALT” | * Please remember to take your medications as prescribed after the survey.  * It is very important that you take your medications as prescribed after we are done. |
|  | Else If “TOOK MEDS” & “NOT MUCH SALT” | * Please remember to weigh yourself after we are done.  * It is important you weigh yourself after you finish the survey. |
|  | Else If “WEIGHED SELF” | * Please remember to take your medications when we are done as prescribed, and to keep your salt intake under 3gms. |
|  | Else If “TOOK MEDS” | * Please remember to weigh yourself and record your weight when we are done, and to comply with the heart failure diet. |
|  | Else | * Please remember to weigh yourself when we are done, and to take your medications as prescribed. |
| Yes | To all: | * “X”, from what you told me, you are experiencing the following symptoms: |
|  | If “WEIGHT INCREASED” | * Your weight has increased more than 3 pounds in the last day. |
|  | If “TIRED REST” | * you feel tired or short of breath when you rest. |
|  | If “SHORT FLAT” | * you feel short of breath when you lie flat in bed. |
|  | If “PILLOW” | * you need to be propped up with pillows in order to be able to sleep without shortness of breath. |
|  | If “SITUP” | * you wake up from sleep in the middle of the night with shortness of breath and need to sit up to feel less short of breath. |
|  | To all: | This is a sign of worsening heart failure. Please notify your heart failure nurse. If this is a medical emergency, please call 911. |
| Yes / No | To all: | Finally, please remember that I am a virtual assistant and not a health care professional. Please use your judgment and seek medical attention if you are not feeling well or have concerns about your health.  * Have a good day and talk tomorrow!  * Good bye, “X”!  * Take care, “X”!  * See you tomorrow.  Good bye “X”!  * Hope you have a good day. Bye! |
