# Supplementary figures and images for: Voice Interface Technology Adoption by Patients With Heart Failure: Pilot Comparison Study
Source: JMIR Mhealth Uhealth. 2021 Apr 1;9(4):e24646. doi: 10.2196/24646 (PMC8050751; doi:10.2196/24646)

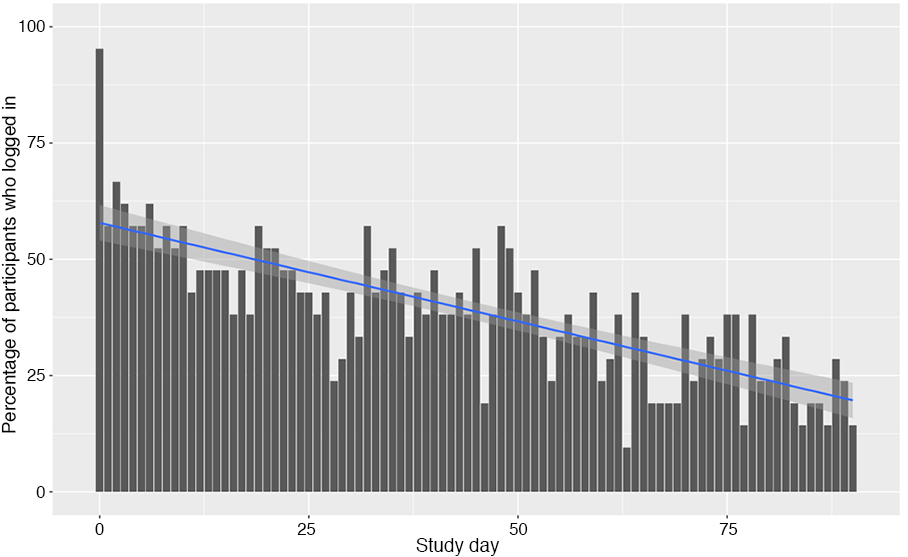

Supplement: Multimedia Appendix 2 [file mhealth_v9i4e24646_app2.png]

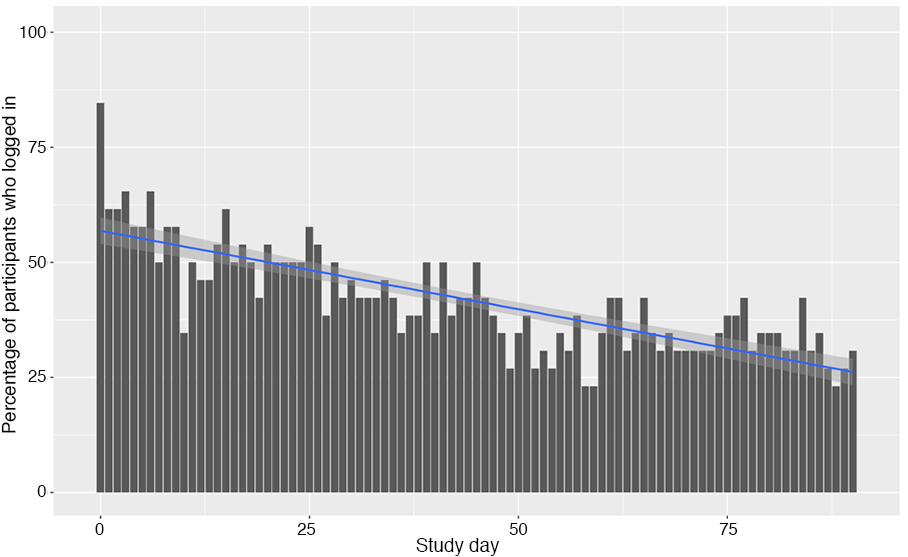

Supplement: Multimedia Appendix 3 [file mhealth_v9i4e24646_app3.png]
